# Remote teaching practices and learning support during COVID-19 lockdowns in Portugal: Were there changes across time?

**DOI:** 10.3389/fpsyg.2022.963367

**Published:** 2022-07-26

**Authors:** Diana Alves, Sofia Marques, Joana Cruz, Sofia Abreu Mendes, Irene Cadime

**Affiliations:** ^1^Centro de Psicologia da Universidade do Porto, Faculdade de Psicologia e Ciências da Educação da Universidade do Porto, Porto, Portugal; ^2^Centro de Investigação em Psicologia para o Desenvolvimento, Instituto de Psicologia e Ciências das Educação, Universidade Lusíada, Porto, Portugal; ^3^Centro de Investigação em Psicologia, Escola de Psicologia, Universidade do Minho, Braga, Portugal

**Keywords:** remote teaching practices, learning support, COVID-19 pandemic, lockdowns, parents' perceptions

## Abstract

The COVID-19 pandemic challenged countries, regions, schools, and individuals. School closures due to lockdowns forced changes in the teaching practices and the learning support provided to children at home. This study aimed to provide insights on the changes between the first and the second lockdowns in Portugal, concerning remote teaching practices and family support to children's education. A self-report questionnaire was filled by 144 parents of third grade students. The results show that, between the two lockdowns, there was a significant decrease in the amount of support provided at home to school assignments and activities, as well as in the amount of time spent by students in TV broadcasted lessons and in reading training supported by the family. Inversely, families reported a significant increase in the amount of time spent by students in independent reading activities and in the time spent in training reading guided by teachers. The number of synchronous lessons with a teacher and the number of times students trained reading during a synchronous lesson also increased in the second lockdown. Additionally, in the second lockdown, parents perceived synchronous lessons to be more effective at improving their child's reading skills and perceived themselves as more capable of supporting their child in reading acquisition. These findings are used to discuss school responses and remote teaching and learning practices during the COVID-19 pandemic.

## Introduction

The COVID-19 pandemic brought an unprecedented disruption in education. To mitigate the transmission and the evolution of the pandemic governments around the world suspended face-to-face teaching in schools, affecting around 95% of the world's student population (United Nations, 2020). As in many other countries, there was a first national lockdown in Portugal starting in March 2020, and elementary school students only returned to school in September of the same year (Ribeiro et al., 2021). Immediate changes were necessary to maintain teaching and to minimize the disruption of students' learning. Emergency Remote Education (ERE), also known as Emergency Remote Teaching and Learning (ERTL), was the response adopted by most countries. ERE should not be understood as a synonym for distance education. Distance education can be defined as a planned and optional method of educational delivery, grounded in theoretical and practical knowledge, whereas ERE is derived from necessity during a crisis and, therefore is unplanned and uses the available resources at the time (Bozkurt and Sharma, 2020; Bozkurt et al., 2020). Thus, at the beginning of the pandemic, ERE led to a shift in activities and curricula planned for face-to-face education to distance environments, without the required planning or ideal conditions, including infrastructure or teacher training. In this scenario, schools offered different packages of support and provision of at distance teaching and home learning guidance for their students (Andrew et al., 2020; Bayrakdar and Guveli, 2020; Pozo et al., 2021; Scully et al., 2021). Schools' responses to school closures ranged from sending worksheets *via* WhatsApp or email, to using Zoom, Google Classroom, Microsoft Teams (or other similar platforms for synchronous remote communication) to host classes and provide assignments (Carvalho et al., 2020; Beach et al., 2021). Some children had online synchronous and asynchronous classes with their teachers, supported by television broadcasted lectures promoted by the government. These measures were not able to reach all pupils, varied across contexts and for some students were delivered with a significant delay (Lindblad et al., 2021; Morgado et al., 2021).

The literature describes four phases in the ERE response during the pandemic: (1) rapid transition to remote teaching and learning; (2) (re)adding the basics; (3) extended transition during continued turmoil; and (4) emerging “new normal” (Barbour et al., 2020). In the first phase, there was a quick switch mainly to synchronous classes online. This was also the case in Portugal, where educational institutions were given 4 weeks to ensure that all regular teaching activities planned for being delivered face-to-face would be transferred to an online learning environment. The Portuguese Ministry of Education provided some support to this transition by creating a website to make materials available and by publishing guidelines for the implementation of distance education at public schools (General Directorate of Education, 2020). Ribeiro et al. (2021) indicate that, in this phase, online classes were a reality for most students in Portugal, as they attended more than 2 h of online classes daily in the first lockdown. However, several difficulties were observed, mainly regarding to the shortage of digital devices, insufficient access to broadband Internet connection, and inadequacy of teachers', parents', and students' digital competences (National Council of Education, 2021). Carvalho et al. (2020) indicate that when changing from face-to-face to remote learning, Portuguese teachers felt difficulties related to the access and use of digital technologies, as well as difficulties in creating innovative materials and delivering them by multiple modalities, and in using creative ways to collaborate with students and parents. In other countries, teachers' lack of proficiency in technology-based teaching, lack of Internet access and/or a personal device were also among the most significant barriers to teaching and learning, during the first lockdown (Scully et al., 2021).

The second phase – (re)adding the basics – implies a greater concern with the quality of the delivered education and with equitable access, compared to phase 1. In Portugal, about 1 month after the beginning of the first lockdown, the government, together with the public television network, provided educational content through television, in a TV program called “Study at Home.” This initiative was created mainly to reach the most isolated student populations which were experiencing difficulties in accessing digital devices and an internet connection. During this period, parents' roles and routines were also forced to change rapidly. The lockdowns led families into new interaction patterns and shaped new roles for parents in their children's education. Childcare and supporting homeschooling were some of the main responsibilities of parents (Fisher et al., 2020; Markowska-Manista and Zakrzewska-oledzka, 2020). Parents needed to invest a significant amount of time to support their children in school activities, suddenly and with little guidance (Andrew et al., 2020), which may have been particularly challenging for parents from lower socio-economic backgrounds (Bayrakdar and Guveli, 2020; Fisher et al., 2020). As reported by Ribeiro et al. (2021), Portuguese parents were involved in supporting their children's study for at least 30 min per day. The same study also indicated that during the first school closure parents supported their children mainly through the monitoring of attention in classes and task realization.

In Portugal, the second lockdown began in January 2021 and lasted for ~2 months. During this period, schools were closed again. As Hodges et al. (2020) highlighted, at distance practices implemented to respond to an unexpected scenario, as it happened in the first lockdown, may be distinct from planned and organized online learning experiences that may have occurred in the second period of school closure. Based on acquired experience, in phase 3 of the ERE response – extended transition during continued turmoil – activities become increasingly more planned and supported by teacher training, infrastructure and equipment, enabling schools to respond more effectively to a new crisis, even if the teaching starts in a face-to-face modality. In Portugal, the research on the practices conducted in the second lockdown is scarcer compared to the first one. However, the experiences of the first lockdown and the measures implemented by schools, local and national authorities, such as providing access to computers or other digital devices to children who did not have it, may have led to changes in the educational practices. Phase 4 of the ERE response – emerging new normal – corresponds to a post-pandemic period in which the level of adoption of online learning can vary. However, the post-pandemic period is out of scope for this work. Instead, this study focuses on the practices adopted in the two lockdowns in Portugal. Its aim was to explore parents' perceptions about changes and challenges related to remote teaching practices and family support to children's education during these two periods.

## Method

### Participants

This study included 144 parents of third grade elementary school students. Students were attending seven public schools in the northern (63.2%), central (34.7%) and southern (2.1%) regions of Portugal (49.3% female, mean age = 7.90 years, SD = 0.42). The questionnaires were responded by 125 mothers (86.8%) and 19 fathers (13.2%), but sociodemographic information regarding both parents in each family was collected. Mothers were aged between 27 and 51 years old (Mean = 39.78; SD = 5.92) and fathers were aged between 28 and 58 years old (Mean = 41.43; SD = 5.85). Regarding maternal education, mothers completed on average 12.71 years of schooling (SD = 4.11), with a minimum of 2 years and a maximum of 20 years. Fathers completed on average 12.78 years of schooling (SD = 3.82), with a minimum of 4 years and a maximum of 20 years of formal education. During the first lockdown, 31.3% of the mothers and 61.1% of the fathers were working on-site, whereas the remainder were at home, working remotely or in a hybrid modality, or without working due to unemployment or other reason (e.g., sick leave). The percentages were similar in the second lockdown, with 26.4% of the mothers and 59.7% of the fathers working on-site.

### Measures

#### Sociodemographic questionnaire

The sociodemographic questionnaire included questions about the parents' individual characteristics (e.g., gender, age, education, working situation) and children (e.g., gender, age and type of school attended).

#### Questionnaire of remote teaching practices and family support

This is a self-report questionnaire developed for purposes of this study to assess parents' perceptions about remote teaching practices and family support during lockdowns. Regarding the remote teaching practices and family support, the questionnaire items collected information on: (1) the number of remote synchronous lessons with a teacher (per week); (2) the number of times the student trained reading aloud in remote synchronous lessons with a teacher (per week); (3) the number of times the student trained reading at home (per week); (4) the frequency of family support in school assignments at home; (5) the frequency that children watched TV broadcasted lessons; (6) the frequency of children's independent reading (reading alone); (7) the frequency that the child practiced reading with the help of a family member; (8) the frequency that the child practiced reading with the help of a teacher. Items 4 to 8 were evaluated through a 4-point Likert scale (1 – never; 2 – rarely; 3 – often; 4 – always). Finally, parents' perceptions on the effectiveness of remote teaching practices and family support during lockdowns were assessed as following: (1) effectiveness of synchronous classes in reading improvement; (2) effectiveness of TV broadcasted lessons in reading improvement; (3) effectiveness of family support in reading improvement; (4) family ability to support their children in reading development. Parents classified each of these items using a four-point Likert response scale, ranging from 1 – not effective/not capable to 4 – very effective/very capable.

### Procedure

Data were collected *via* a web-survey whose link was sent to parents via email. In the first moment of data collection (September – October 2020), parents were asked to report their experience retrospectively, given that the first lockdown had already finished. In the second moment of data collection (February – March 2021), parents were asked to answer to the same items based on their current experiences during the second lockdown. The study was approved by the ethics committee of Lusíada University, following the guidelines of the Psychology for Positive Development Research Center (CIPD/2122/DEED/1), and was part of a larger research project focused on reading skills of third graders (Cruz et al., 2022). The study was presented to parents by their children's teachers. For the parents who participated in the study in the first moment of data collection, an email was sent asking again for their collaboration in the study during the second lockdown. The participation was voluntary, with no incentives for participation being offered. The confidentiality and the anonymity of all data were guaranteed. The completion of the questionnaires took between 10 and 12 min.

### Data analysis

To test for differences between lockdowns in the metric variables, paired-samples *t*-tests were performed. Cohen's *d* was used as a measure of effect size: 0.20 suggests a small, 0.50 a medium, and 0.80 a large effect (Cohen, 1988). To test for the differences in the ordinal variables, the Wilcoxon signed-rank test was used. For this test, the *r* value was calculated as a measure of effect size. According to Cohen (1988) *r* values above 0.1 can be described as small, above 0.3 can be described as medium, and values above 0.5 can be described as large.

## Results

Table 1 presents the results of the Wilcoxon tests for the change in the practices and learning support provided to children between the two lockdowns. The results suggest a small decrease in the amount of support in school assignments provided at home by family members and in the reading training supported by the family. The results also indicate a large decrease in the amount of time spent by students in TV broadcasted lessons in the second lockdown, compared to the first one. On the contrary, the frequency of independent reading by the child and the frequency of reading training supported by teachers at a distance, increased significantly in the second lockdown.

**Table 1:** Parents' perceptions practices and learning support during the remote teaching in the first and second lockdowns.

	**1st lockdown**			**2nd lockdown**			***n***	**z**	***p***	***r***
**Items**	**P25**	**Mdn**	**P75**	**P25**	**Mdn**	**P75**				
The child was supported by a family member in school assignments at home	4.00	4.00	4.00	3.00	4.00	4.00	144	−3.762^b^	<0.001	0.22
The child watched TV broadcasted lessons	3.00	4.00	4.00	2.00	2.00	3.00	143	−8.875^b^	<0.001	0.52
The child practiced reading alone (independent reading)	3.00	3.00	3.00	3.00	3.00	4.00	140	−2.592^a^	0.010	0.15
The child practiced reading with the help of a family member	3.00	3.00	4.00	2.00	3.00	3.00	139	−2.975^b^	0.003	0.18
The child practiced reading with the help of a teacher, remotely	1.00	2.00	3.00	3.00	3.00	3.00	135	−6.333^a^	<0.001	0.39

P25, 25^th^ percentile; P75, 75^th^ percentile; Mdn, median; ^a^Based on negative ranks; ^b^Based on positive ranks. Likert scale: 1 – never; 2 – rarely; 3 – often; 4 – always.

Table 2 presents the results of the paired-samples *t*-tests to explore the changes in the number of remote synchronous lessons and reading practice between the two lockdowns. The number of remote synchronous lessons with a teacher and the number of times students trained reading during a synchronous lesson increased significantly in the second lockdown, compared to the first one. In both cases, the effect size was large. There was no significant difference in the number of times the student trained reading at home between the two lockdowns.

**Table 2:** Number of synchronous school lessons and reading practice in the two lockdowns.

	**1st lockdown**	**2nd lockdown**	***n***	***t(df)***	***p***	***d***
**Items**	**Mean (SD)**	**Mean (SD)**				
Number of remote synchronous lessons with a teacher (per week)	1.61 (0.850)	6.69 (5.282)	121	−10.728 (120)	<0.001	1.34
Number of times the student trained reading aloud in remote synchronous lessons with a teacher (per week)	1.25 (1.100)	2.85 (3.333)	130	−5.285 (129)	<0.001	0.64
Number of times the student trained reading at home (per week)	3.81 (1.957)	4.03 (2.553)	99	−0.853 (98)	0.396	0.09

SD, standard deviation; df, degrees of freedom.

Table 3 presents the results of the Wilcoxon tests to explore the differences in parents' perceptions of the efficacy of the practices and learning support provided to children during remote teaching. The results indicate that, in the second lockdown, parents perceived synchronous lessons to be more effective at improving their child's reading skills and perceived themselves as more capable of supporting their child in reading acquisition. The effect size of this increase in the perceived capability was large. The results also suggest a small decrease in the perceived effectiveness of TV broadcasted lessons in improving children's reading skill.

**Table 3:** Parents' perceptions on the efficacy of the practices and learning support in children's reading skills during the remote teaching.

	**1st lockdown**			**2nd lockdown**			***n***	***z***	***p***	***r***
**Items**	**P25**	**Mdn**	**P75**	**P25**	**Mdn**	**P75**				
Effectiveness of synchronous classes in reading improvement	2.00	3.00	3.00	3.00	3.00	4.00	131	−4.222^a^	<0.001	0.26
Effectiveness of tv broadcasted lessons in reading improvement	2.00	2.00	3.00	1.00	2.00	2.00	129	−4.213^b^	<0.001	0.26
Effectiveness of family support in reading improvement	3.00	3.00	4.00	3.00	3.00	4.00	138	−0.503^b^	0.615	0.03
Parents' perception of ability to support their children in reading development	2.00	2.00	3.00	3.00	4.00	4.00	144	−10.363^a^	<0.001	0.61

P25, 25^th^ percentile; P75, 75^th^ percentile; Mdn, median; ^a^Based on negative ranks; ^b^Based on positive ranks. Likert scale ranging from 1 (not effective/not capable) to 4 (very effective/very capable).

## Discussion

In the last 2 years, research intensively investigated the effects of school lockdowns due to the COVID-19 pandemic on involved stakeholders, such as teachers, students, and parents. However, as research projects had to be hurriedly conducted, in-depth and longitudinal studies are lacking (Lindner et al., 2021). The goal of this study was to explore parents' perceptions about changes in the remote teaching practices and family support provided to children during the first and the second lockdowns in Portugal, in a longitudinal study with two points of data collection. These were challenging times for parents, as most of them had to combine the supervision of children's school lessons and assignments while trying to work from home (Morse et al., 2022; Seabra et al., 2022). Moreover, for parents who were not working from home, going back and forth to work with concerns about potential contamination of their homes were additional sources of stress (Anderson et al., 2021). Nonetheless, parents are privileged informants on the educational practices conducted at home during these periods.

The results of this study suggest that in the second lockdown there was a small decrease in the amount of support provided at home by family members in school assignments and in assisting students' reading training, as well as a large decrease in the amount of time spent by students in TV broadcasted lessons. At the same time, the number of remote synchronous lessons with a teacher increased significantly in the second lockdown. As indicated before, the first lockdown was characterized firstly, by a rapid and unplanned transition to remote teaching and learning, and secondly, by the creation of additional educational responses to guarantee accessibility of all students, of which the best example was the creation of the “Study at Home” TV program. These responses correspond to the first two phases of the ERE response during the pandemic (Barbour et al., 2020). This reality was not only verified in Portugal, but also in other countries (Gunzenhauser et al., 2021; Lindner et al., 2021).

In these initial phases, teachers were faced with unique challenges in adapting to remote education with little planning and with variable resources, experience, and training (Carvalho et al., 2020; Scully et al., 2021). Overall, the findings of this study suggest that in the second lockdown, Portugal was already in phase 3 of the ERE – extended transition during continued turmoil (Barbour et al., 2020). The time between the two lockdowns probably allowed schools and local and national authorities to better address the necessities in terms of infrastructures and equipment, as well as in investing in teacher training for remote education, thus leading to a more consolidated response. This idea is also reinforced by the results of our study that show a significant increase in the number of times students trained reading during a synchronous lesson and in the frequency of reading training supported by teachers at a distance in the second lockdown. These findings suggest that the online learning experiences were more planned and organized in the second lockdown, as suggested by Hodges et al. (2020). The development of reading skills is a central goal in the 1st years of schooling and therefore, the increase in the number of synchronous sessions probably allowed more time for these essential skills to be directly targeted.

Overall, the findings of this study suggest that the amount of support required from parents decreased, whereas the support given by teachers increased between the two lockdowns. This is an important conclusion, given that some concerns have been raised that by making the learning process rely more on families, rather than on teachers during lockdowns, social class discrepancies could be exacerbated (Goudeau et al., 2021). A complementary finding was that not only the frequency of reading training supported by teachers increased, but also the frequency of independent reading by the child alone. According to the Self-determination theory (Ryan and Deci, 2000) autonomous motivation to engage in activities is dependent on conditions or teaching dimensions supporting the students to meet their basic psychological needs for autonomy, competence, and relatedness. Conditions or teaching dimensions considered relevant here are the autonomy support that refers to giving students age-appropriate choices, offering rationales, taking the students' perspective and providing them with opportunities to take the initiative during classes, structure which concerns to communicating expectations clearly, offering help and support, responding consistently and providing feedback, and involvement that is related to the quality of the relationship between teachers and peers and the investment they put in Reeve (2002). Although these teaching dimensions were not formally evaluated in our study, the more planned and organized online learning experience in the second lockdown, along with the existence of more moments of synchronous classes, more number of times students trained reading during a synchronous lesson and the increase in the frequency of reading training supported by teachers at a distance, may well have contributed to enhance the support for autonomy, structure and involvement that students need to potentiate their autonomous motivation for independent reading.

Regarding the perceived efficacy of remote teaching practices, in the second lockdown, parents perceived synchronous lessons to be more effective at improving their children's reading skills, along with a small decrease in the perceived effectiveness of TV broadcasted lessons in improving children's reading skills. On the one hand, these findings cannot be dissociated from the reported increase in the number of synchronous lessons with teachers and decrease of frequency of watching TV broadcasted lessons. On the other hand, these findings can also be a result of the more organized learning activities during the second lockdown. As Vygotsky (1978) indicated, social interactions are a crucial context for student learning (Erbil, 2020) and these occur in synchronous lessons but not when students receive information from the TV. In the first lockdown, for most Portuguese teachers, the synchronous sessions were an effective way to deliver content rapidly, without having the time, the means, or the knowledge to apply a more elaborate learning design. In fact, few teachers used synchronous sessions to promote discussion, interaction, and socialization during that period (National Council of Education, 2021). It is possible that the design of the sessions improved during the second lockdown, but more studies are needed to support this hypothesis.

Another finding of this study was that parents perceived themselves as more capable of supporting their child in reading acquisition in the second lockdown compared to the first one. Parental monitoring during the COVID-19 lockdowns might not be directly comparable to parental monitoring in regular homework situations, because parents might have felt a greater responsibility to oversee their child's schoolwork in a situation where no teacher was present on a regular basis (Gunzenhauser et al., 2021). However, as the online presence of teachers increased in the second lockdown, parents may have felt more supported and therefore more capable to monitor and help their children in developing essential skills such as reading. Additionally, it is probable that parents already had learned how to use more effectively the tools employed in remote education, and that the communication between them and the schools had increased in the second lockdown, thus leading to an increased sense of self-efficacy. Future studies could explore these issues.

In conclusion, these findings suggest a progressive adjustment of schools and families to the remote teaching practices across time, after the initial rapid and unplanned transition from the face-to-face to the remote modality. As Bozkurt et al. (2020) suggest, in the more advanced phases of ERE, schools and other educational authorities should “continue to improve the current teaching and learning experience, prepare for the eventual post-COVID future, and maintain a state of readiness for future school closures requiring rapid shifts to remote teaching and learning” (p. 12). As we seem to approach the “new normal,” this is the time to use the previous experience to make remote education more effective in future crises (e.g., pandemics, war scenarios) that can impact any population and preclude children from going to school. Moreover, the investment made in equipment such as computers and appropriate software, and the acquired experience by all stakeholders can also make remote education a feasible solution to other problems – for example, to reach children who are hospitalized for long periods of time. The availability of these resources can also be an opportunity to make access to knowledge easier and thus reduce the inequalities between children from different socioeconomic backgrounds, in what seems to be an opportunity that arose from a global crisis.

### Limitations and future studies

Despite the relevance of the current results, the generalization of the findings should consider some limitations. The sample is not representative of the population; thus, caution is recommended when inferences are drawn. Also, teachers were not asked to answer the questionnaire regarding remote teaching practices and family support. We rely only on parents' answers, while recognizing that having multiple informants about teaching practices and family support would have been valuable for this study. We should also consider that parents answered the questionnaire regarding the first lockdown in a retrospective way, more prone to recall bias and to some inaccuracy on data reported. We also do not have data regarding the practices in the pre-pandemic period, which could allow us to draw richer conclusions about change. In future studies, the findings of our study can also be complemented with qualitative data to further explore parents view on education during this period.

## Data availability statement

The raw data supporting the conclusions of this article will be made available by the authors, without undue reservation.

## Ethics statement

The studies involving human participants were reviewed and approved by the Ethics Committee of Lusíada University, following the guidelines of the Psychology for Positive Development Research Center (CIPD/2122/DEED/1). The patients/participants provided their written informed consent to participate in this study.

## Author contributions

DA, SM, JC, and SAM made substantial contributions to conception and design of the study, data collection, and discussion of the results. IC made substantial contributions to conception and design of the study, statistical data analysis, and discussion of the results. All authors were involved in drafting the manuscript and revising it critically for important intellectual content.

## Funding

This work was financially supported by the Portuguese Foundation for Science (FCT) and Technology and the Portuguese Ministry of Science, Technology, and Higher Education through national funds within the framework of the Psychology for Positive Development Research Center–CIPD (grant number UIDB/04375/2020) and the Psychology Research Centre (UIDB/PSI/01662/2020). The last author (IC) was also supported by the Foundation for Science and Technology (CEECIND/00408/2018).

## Conflict of interest

The authors declare that the research was conducted in the absence of any commercial or financial relationships that could be construed as a potential conflict of interest.

## Publisher's note

All claims expressed in this article are solely those of the authors and do not necessarily represent those of their affiliated organizations, or those of the publisher, the editors and the reviewers. Any product that may be evaluated in this article, or claim that may be made by its manufacturer, is not guaranteed or endorsed by the publisher.
